# Effectiveness of fully immersive virtual reality-based simulation training on objective knowledge acquisition in acute coronary syndrome/ST-elevation myocardial infarction emergency management: a pre-post-intervention study

**DOI:** 10.1093/ehjdh/ztaf094

**Published:** 2025-09-04

**Authors:** Jonas Einloft, Philipp Russ, Simon Bedenbender, Hendrik L Meyer, Muriel L Morgenschweis, Andre Ganser, Andreas Jerrentrup, Martin C Hirsch, Ivica Grgic

**Affiliations:** Department of Internal Medicine and Nephrology, University Hospital Giessen and Marburg, Marburg University, Baldingerstraße, Marburg 35043, Germany; Department of Internal Medicine and Nephrology, University Hospital Giessen and Marburg, Marburg University, Baldingerstraße, Marburg 35043, Germany; Institute for Artificial Intelligence in Medicine, University Hospital Giessen and Marburg, Marburg University, Baldingerstraße, Marburg 35043, Germany; Department of Internal Medicine and Nephrology, University Hospital Giessen and Marburg, Marburg University, Baldingerstraße, Marburg 35043, Germany; Department of Internal Medicine and Nephrology, University Hospital Giessen and Marburg, Marburg University, Baldingerstraße, Marburg 35043, Germany; Department of Internal Medicine and Nephrology, University Hospital Giessen and Marburg, Marburg University, Baldingerstraße, Marburg 35043, Germany; Department of Internal Medicine and Nephrology, University Hospital Giessen and Marburg, Marburg University, Baldingerstraße, Marburg 35043, Germany; Department of Emergency Medicine, University Hospital Giessen and Marburg, Marburg University, Baldingerstraße, Marburg 35043, Germany; Institute for Artificial Intelligence in Medicine, University Hospital Giessen and Marburg, Marburg University, Baldingerstraße, Marburg 35043, Germany; Department of Internal Medicine and Nephrology, University Hospital Giessen and Marburg, Marburg University, Baldingerstraße, Marburg 35043, Germany; Institute for Artificial Intelligence in Medicine, University Hospital Giessen and Marburg, Marburg University, Baldingerstraße, Marburg 35043, Germany

**Keywords:** Acute coronary syndrome, STEMI, Emergency management, Immersive technologies, VR-based simulation training, Preparedness for real-life scenarios

## Abstract

**Aims:**

Effective management of emergencies, particularly acute coronary syndrome (ACS), demands rapid, guideline-based interventions to optimize outcomes. However, many medical students and young professionals report feeling unprepared due to limited hands-on experience. Virtual reality (VR) presents a promising training tool, though its efficacy remains unproven.

**Methods and results:**

In this single-center study, 247 medical students were assigned to three different guidance modes to manage a virtual ST-elevation myocardial infarction patient using the Simulation-based Training of Emergencies for Physicians using Virtual Reality (STEP-VR) application. A pre-post-test design, based on European Society of Cardiology (ESC) guidelines, was used to evaluate learning outcomes. Our results showed a significant increase in knowledge after the training. Students in the tutor-moderated ‘human guidance’ group demonstrated the greatest knowledge improvement (M=+24%,SD=13%), being significantly better than the ‘no guidance’ group (M=14%, SD=9%). However, there was no significant difference between the ‘human guidance’ group and the ‘integrated guidance’ group (M=+19%,SD=14%), which used an embedded learning mode within STEP-VR. To evaluate the potential impact on clinical performance, we calculated composite quality indicators based on ESC-defined metrics. Consistently, we found a significant improvement in these indicators [clinical quality indicators (CQI) 0.47 (pre) vs. 0.76 (post) and 0.8 (post), respectively], with no significant difference between the ‘human guidance’ and ‘integrated guidance’ groups.

**Conclusion:**

In conclusion, our findings demonstrate that VR-based acute coronary syndrome/ST-elevation myocardial infarction training is both operationally feasible and educationally effective. Notably, integrated guidance yielded outcomes comparable to tutor-led instruction, underscoring the potential of this approach as a platform for independent, extracurricular learning. While our data suggest VR training may support clinical performance, future studies with objective assessments are needed to confirm its real-world value.

## Graphical Abstract

**Graphical Abstract:**
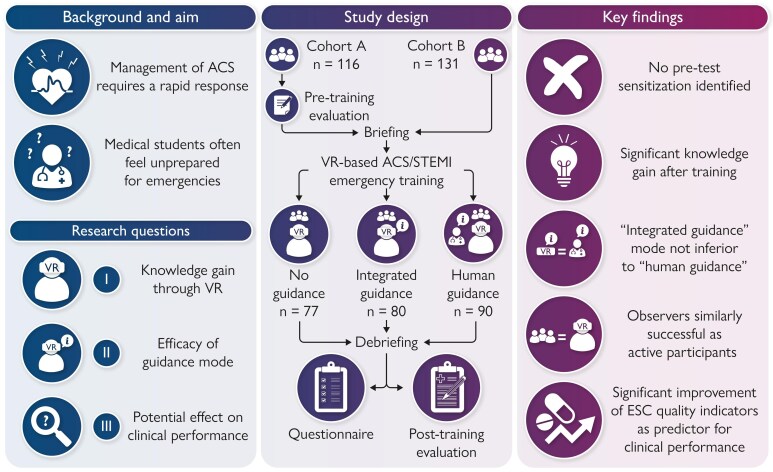

## Introduction

Cardiovascular diseases (CVD), including coronary artery disease (CAD) and its major clinical manifestation acute coronary syndrome (ACS), remain a leading cause of death, accounting for approximately 17.9 million deaths in 2019—nearly one-third of all global fatalities.^1^

ACS is classified into unstable angina, non-ST-segment elevation (NSTEMI) and ST-segment elevation (STEMI), with the latter accounting for approximately 30% of all ACS cases.^2^ In emergency medical management, timely intervention is critical, as studies have demonstrated a positive correlation between rapid reperfusion and reduced mortality.^3^ To optimize patient care, the European Society of Cardiology (ESC) has published evidence-based guidelines for the management of STEMI and NSTEMI in 2017,^4^ with an updated version released in 2023.^5^

Despite well-established STEMI management protocols, a significant gap persists between recommended guidelines and actual clinical practice.^6,7^ Alarmingly, many medical students and young residents report feeling unprepared to handle emergencies due to insufficient hands-on practice.^8–10^ Research by McEvoy *et al*.^11^ found that fourth-year medical students performed significantly worse in managing STEMI patients compared to those with stable conditions. Moreover, effective management of emergencies requires strong clinical reasoning skills to ensure competent medical care.^12^ To address these deficiencies, simulation-based medical education (SBME) has emerged as a proven modality for enhancing clinical skills, decision-making, and confidence in managing acute medical conditions, particularly in cardiology.^13,14^

Despite the well-documented benefits of SBME, its widespread adoption is often limited by substantial barriers, including the need for extensive resources with associated high costs as well as the need for specialized facilities and trained personnel.^15,16^ Additionally, these simulations have limited scalability preventing the training of a larger number of students. These limitations highlight the necessity for innovative, cost-effective training solutions that can be scaled and adapted to diverse learning environments.

Virtual reality (VR) technology offers a compelling alternative to traditional simulation models by providing immersive, interactive experiences that closely replicate real-life clinical scenarios. Furthermore, VR-based training allows learners to practice managing complex medical emergencies in a controlled, risk-free environment, fostering experiential learning and skill development. Additionally, the flexibility and accessibility of VR enable personalized learning pathways tailored to individual skill levels and learning preferences.^17–19^

We recently observed a high level of acceptance for the use of VR in medical education among both students and educators. Notably, when asked about potential applications, students frequently mentioned emergency medicine as a promising area.^20^ Based on this, we tested a novel VR-based training software for medical emergencies, ‘Simulation-based Training of Emergencies for Physicians using Virtual Reality’ (STEP-VR), which was recently developed by the University Hospital of Würzburg in cooperation with ThreeDee GmbH.^21,22^ Importantly, the curricular implementation of STEP-VR was found to be feasible and well-accepted by students.^20,23^ Initially, reported learning success was based solely on subjective assessments, emphasizing the need for further research. Emerging evidence suggests that these applications may lead not only to short-term knowledge gains but may also offer long-term learning advantages and sustained long-term learning gains.^24^ However, the optimal design of the associated course structure remains to be determined. Moreover, considering clinical quality indicators (CQIs) in addition may add a valuable, application-focused dimension to the assessment. Such indicators capture not only the acquisition of theoretical knowledge but also its appropriate integration and prioritization within clinical decision-making, thereby offering a more comprehensive measure of clinical reasoning and adherence to guidelines in simulated emergency scenarios.

In this study, we aimed to refine our analysis by focusing exclusively on a single high-stakes emergency scenario: the management of a patient presenting with ACS. Specifically, we sought to address the following key questions:

- Does VR-based training for ACS management lead to significant improvements in both subjective and objective knowledge acquisition?
- Which seminar design is most effective in maximizing learning outcomes within the context of ACS training?
- Could this educational approach contribute to measurable enhancements in the quality of care, as assessed by established CQIs?

## Methods

### Survey participants

To assess the educational potential of VR-based management of ACS emergencies, we conducted a study with fifth-year medical students at Philipps University of Marburg. The immersive training was integrated into the curricular practical course in internal medicine, designed for senior medical students.

### Virtual reality-head-mounted display and virtual reality application

In this study, we used the commercially available Oculus Quest 2 (now known as Meta Quest 2) head-mounted displays (HMDs), which offer portability, inside-out tracking, and wireless connectivity to the host computing device, enabling unrestricted movement. As the host computing device, we used Acer Predator Helios 300 laptops equipped with an Intel Core i7-10750H CPU, NVIDIA GeForce RTX 3080 Laptop GPU (8 GB VRAM), and 32 GB DDR5 RAM. Wireless streaming to the VR-HMD was conducted via Meta Quest Air Link over an Asus AX5400 router.

For emergency room simulation, we employed the STEP-VR application developed by ThreeDee GmbH (Munich, Germany) in collaboration with the Medical School of the University of Würzburg.^21,22^ STEP-VR is specifically designed for interactive simulation training in medicine and features a variety of independent emergency scenarios. The simulation allows for patient assessment and diagnostics, including medical history taking, electrocardiograms (ECG), circulatory and pulse oximetry monitoring, standard radiology, and blood testing. Additionally, the simulation enables the initiation of expected interventions, such as oxygen and drug administration, as well as treatments like heart catheterization and endoscopy. Interactive procedures are performed in a simplified manner to facilitate learning and engagement.

### Acute coronary syndrome/ST-elevation myocardial infarction simulation scenario

**Figure 1:**
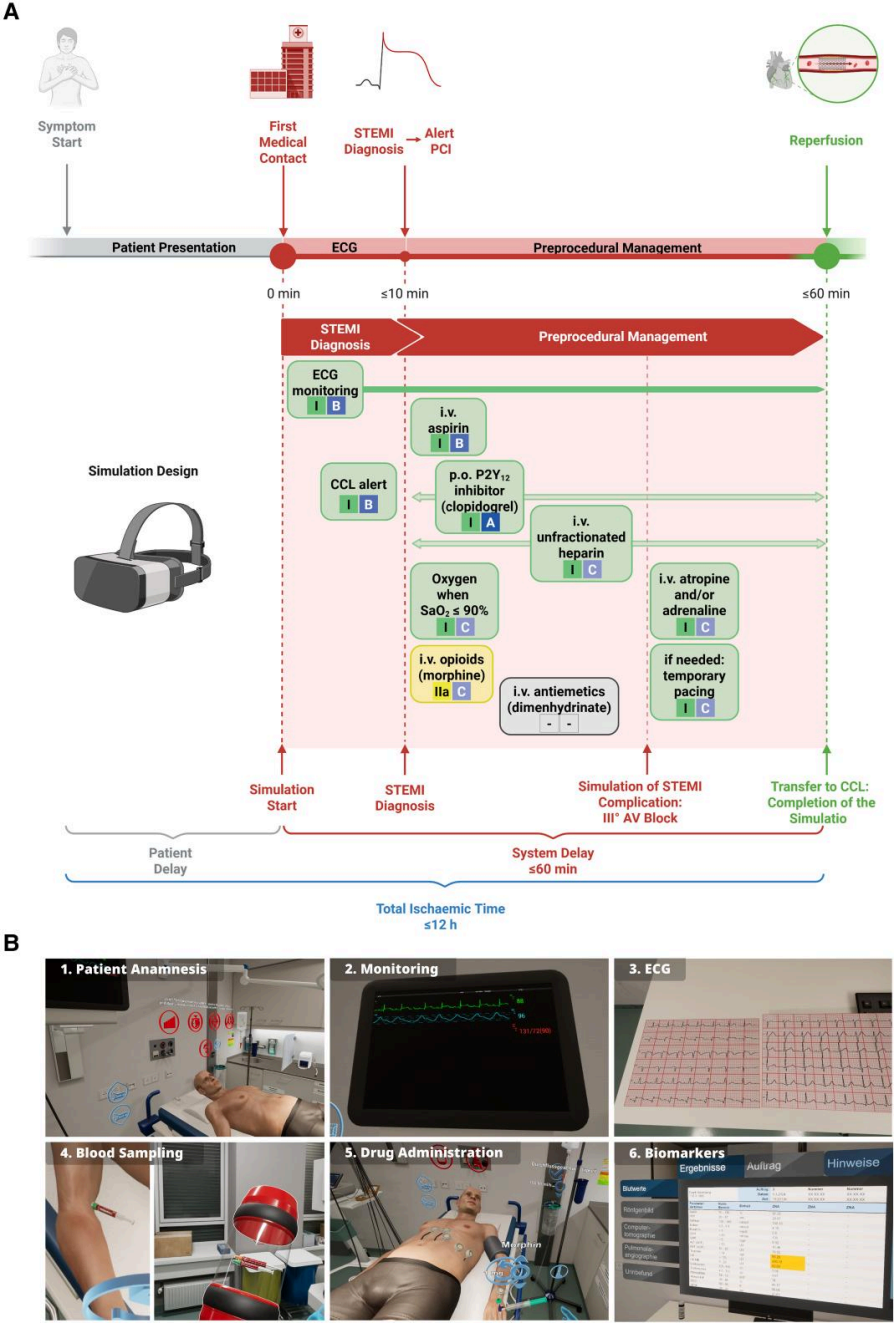
Implementation of fully immersive ACS/STEMI simulation training in a virtual emergency room setting. (*A*) Illustration highlighting the alignment of the 2017 ESC guideline recommendations with the corresponding tasks integrated into the simulation. (*B*) In-simulation first-person views showcasing the implemented tasks and key features. Created in BioRender.

In this study, students were confronted with a medical emergency scenario without prior knowledge of the case type. As a use case, we selected ACS—a prototypical cardiological emergency—which was included among the pre-existing scenarios in the STEP-VR application. Specifically, students managed a STEMI case involving a male patient in his mid-50s, who presented with severe retrosternal pain that first occurred during nocturnal sleep. The case implementation followed the 2017 ESC guidelines for the management of acute myocardial infarction in patients presenting with ST-segment elevation and the 2023 ESC guidelines for the management of acute coronary syndromes.^4,5^ As the first medical contact (FMC) in the simulation occurs in the emergency department, participants were required to diagnose STEMI within 10 min and achieve successful reperfusion within 60 min, as per ESC recommendations (*Figure 1A*). To meet these requirements, trainees were expected to promptly initiate ECG monitoring, including a detailed 12-lead ECG, immediately after identifying key clinical cues in the patient’s medical history. This would enable them to confirm STEMI within the 10 min window and promptly alert the cardiac catheterization laboratory. The diagnosis could be further corroborated by blood sampling to assess cardiac biomarkers (troponin, CK, CK-MB). After confirming the diagnosis, students were tasked with providing the appropriate antithrombotic therapy, as well as administering antiemetic and analgesic treatment when necessary. Oxygen therapy was only to be given if SpO₂ dropped below 90%. Subsequently, a sudden third-degree AV block is simulated (see Supplementary material online, *Figure S1*). At this stage, students were required to immediately apply an external defibrillator for potential pacing and administer atropine to manage bradyarrhythmia. A summary of the implemented treatments, including their evidence and recommendation levels, is provided in *Figure 1A*. Screenshots illustrating the corresponding simulation implementation can be found in *Figure 1B* and Supplementary material online, *Figure S1*. After completing the scenario, students received an evaluation of their performance based on their achievement of key medical goals. Additionally, a visual representation of treatment progress and corresponding parameters was provided (see Supplementary material online, *Figure S1*).

### Study design

**Figure 2:**
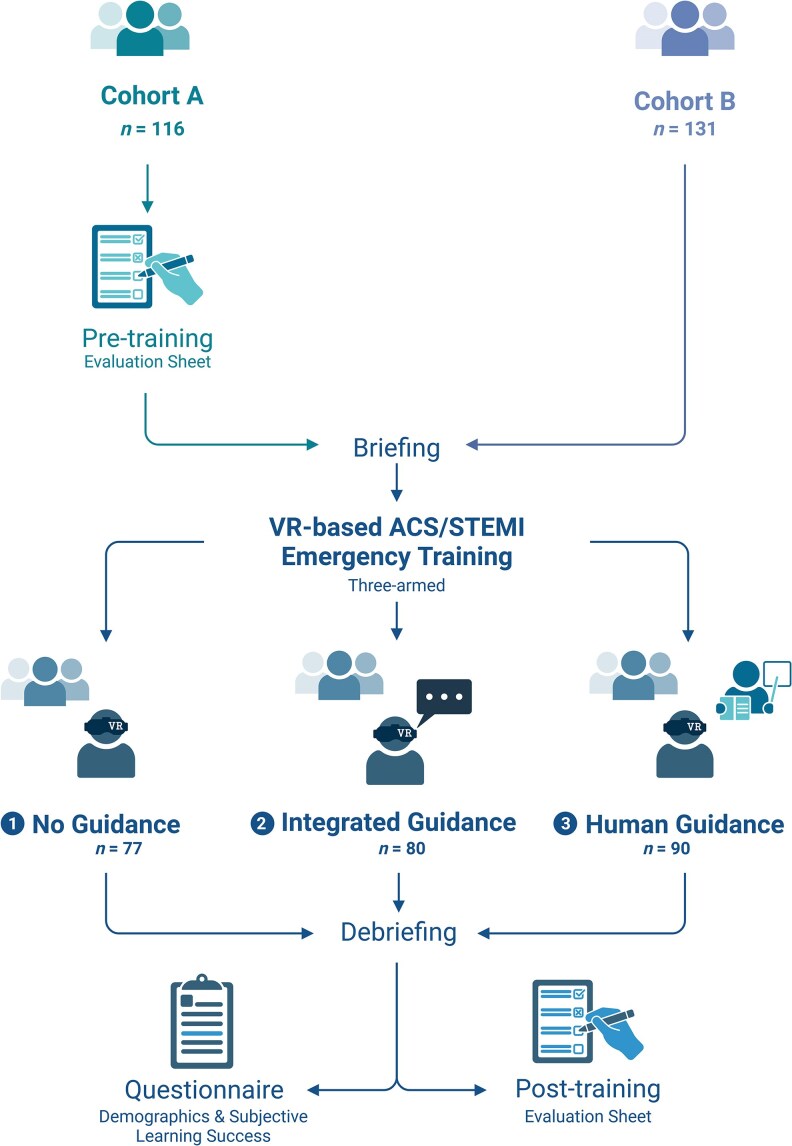
Study design and flow chart. A total of 247 students participated in two cohorts. Cohort A completed the evaluation sheet both before and after the training, while Cohort B only completed it post-training without prior exposure to the questions. All students were randomly assigned to one of three guidance modes: (i) no guidance, (ii) integrated guidance, or (iii) human guidance. Following the debriefing, all students filled in the questionnaire and the post-training evaluation sheet. Created in BioRender.

The VR-based emergency training was conducted as peer-group seminars led by a student tutor, forming part of the practical course in internal medicine for fifth-year medical students (*Figure 2*). The total duration of each seminar was approximately 1.5 h.

Students were randomly assigned by the organizing team into groups of 6–8 participants and placed into one of three seminar modes:

• No guidance—students completed the seminar independently, without medical input from the tutor. To ensure unbiased allocation, the three available seminar formats were written on slips of paper, corresponding to the number of seminar sessions, and randomly drawn in a blinded process. The tutor only provided technical support in case of VR-related issues.

• Integrated guidance—the tutor remained available for technical support, but students utilized the built-in Learning Mode of STEP-VR. This mode provided in-game hints and time-triggered prompts if required treatments were not initiated or if vital parameters exceeded or fell below specific thresholds.

• Human guidance—the tutor acted as both a technical assistant and seminar moderator, providing medical guidance throughout the session. This design allowed for a comparative analysis of different levels of guidance in VR-based ACS/STEMI management training.

The seminars started with a briefing session, which included an introduction to the VR device and software. To familiarize participants with the simulation, they first completed a medical emergency case unrelated to cardiac conditions before proceeding to the ACS/STEMI case, as described earlier. During the seminar, one student actively participated (AP) by wearing the VR device and managing the case, while the remaining students acted as observers (OBS), following the simulation on a screen and collaboratively discussing the diagnosis and treatment approach. Participants in a group were free to rotate roles as the case progressed. The individual exposure time in VR varied, ranging from approximately 10 to a maximum of 30 min per participant, depending on group size and rotation. After completion of the VR simulation, the tutor concluded the session with a concise debriefing focusing on the immersive experience of the students. It was ensured that any discussion of ACS/STEMI-specific content or learning objectives assessed in the post-intervention questionnaire was avoided. Students were then asked to complete (i) a questionnaire covering demographic information and three questions related to subjective learning design (see Supplementary material online, *Table S1*) and (ii) an adapted 10-question test assessing key knowledge on ACS/STEMI diagnosis and treatment, based on the 2017 and 2023 ESC guidelines,^4,5,24^ which served as a measure of learning success (see Supplementary material online, *Table S2*). A detailed summary of the 10 questions, including scoring and ESC class/level, is provided in Supplementary material online, *Table S2*. Scoring was performed by a single, non-blinded rater following standard procedures used in exam grading based on the criteria presented in Supplementary material online, *Table S2*. A second rater independently verified a random sample of the ratings to ensure consistency.

For Cohort A, the questionnaire was handed to students at the very beginning of the seminar to assess students’ pre-training knowledge and then again at the end of the seminar to evaluate learning success. For Cohort B, the questionnaire was only provided after the training to determine whether exposure to the questions influenced students’ responses, thereby assessing potential bias (*Figure 2*). The study protocol was not published or registered prior to the commencement of the study.

### Data analysis

The survey data, obtained through printed close-ended and open-ended questions, were analysed using the statistical software R (4.3.0) and RStudio (2023.12.1). Responses were manually digitized and entered into an Excel spreadsheet, which served as input for data analysis. Demographic statistics including age, gender, study semester, previous VR experience, and experience with first-person games were calculated based on the questionnaire data. Figures were generated using the ggplot2 (3.4.3) and the ggpubr (0.6.0) R packages. Likert-scale questions assessing subjective learning success were analysed using the Likert (1.3.5) R package, and Likert plots for each question were created using the ggplot2 (3.4.3).

Objective learning success was defined as the improvement in total score after the VR-based training, calculated as the difference between total scores before and after training (using data exclusively from Cohort A). Differences in learning success between seminar modes and participation roles were statistically analysed using the Mann–Whitney *U* test, as normality could not be assumed according to Shapiro–Wilk testing. Calculation was performed with the *pairwise.wilcox.test* function from the stats (4.3.0) R package. Results were considered statistically significant for α≤0.05.

The calculation of quality indicators (QIs) was based on the definitions provided by the ESC.^4,25,26^ The defined measures were applied to the evaluation sheet data to derive the corresponding QIs. Among the various QIs and composite QIs (CQIs) developed, the opportunity-based CQI shows a strong link to adjusted mortality and effectively differentiates levels of care. As a result, our attention was directed towards CQIs. In brief, the opportunity-based CQI considers the number of treatments received out of the total number of potential treatments (‘opportunities’) for a certain patient. In contrast, the all-or-none CQI only considers the number of patients who received all applicable treatments (all-or-none principle). A detailed description of the QI calculation is provided in Supplementary material online, *Table S3* (for individual QIs) and Supplementary material online, *Table S4* (for composite QIs, CQIs). Statistical testing was performed using the Mann–Whitney *U* test for opportunity-based CQI, as normality could not be assumed according to Shapiro–Wilk testing. Calculation was implemented with the *pairwise.wilcox.test* function from the stats (4.3.0) R package. Fisher’s exact test was applied for all-or-none CQI, performed with the *pairwise.fisher.test* function from the reporttols (1.1.3) R package.

### Ethics approval

An exemption from ethics approval was granted by the Ethics Committee of the Medical Faculty of Marburg University, as no approval was required according to the German Code of Medical Ethics (file number ‘23–129’).

## Results

### Demographics

**Figure 3:**
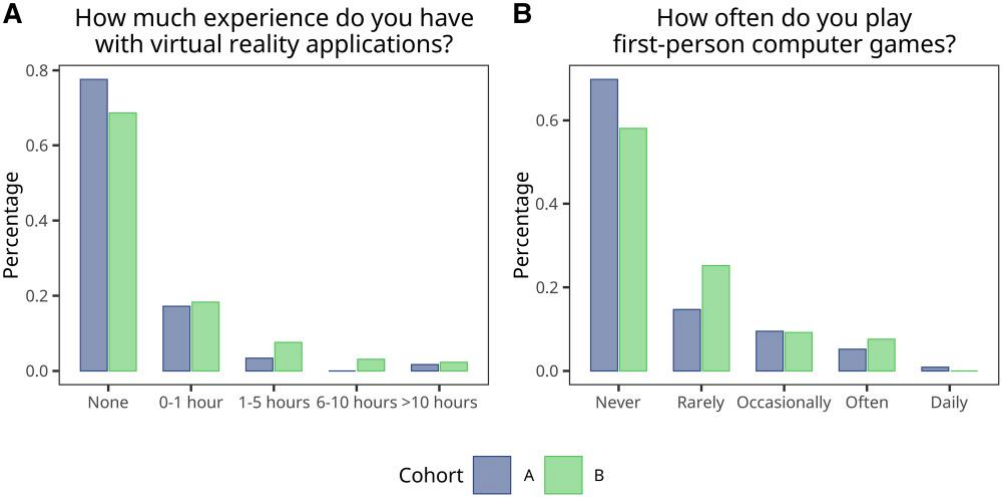
Descriptive plots of reported prior VR experience (*A*) and frequency of first-person computer game play (*B*). Bars represent percentages relative to the total study population.

Our study included a total of 247 medical students, of whom 168 (68%) identified as female and 78 (31.6%) as male. Their ages ranged from 22 to 36 years, with an average age of 25 and a median of 24 years (*Table 1*). The participants were divided into two cohorts, with 116 students (47%) assigned to Cohort A and 131 students (53%) to Cohort B. Notably, the majority of students had no prior experience with VR technology before taking part in the study, and most did not regularly play first-person computer games (*Figure 3*). Regarding seminar participation, students were distributed across three different modes: 77 students (31.2%) attended without guidance, 80 students (32.4%) participated using the built-in learning mode, and 90 students (36.4%) were in the tutor-guided sessions. Regarding participation roles, 76 students (30.8%) actively immersed themselves in the simulation, wearing the VR device and managing the case. The remaining 171 students (69.2%) observed the scenario as it unfolded, collaboratively offering insights and contributing to decision-making discussions. A detailed summary of demographic characteristics is provided in *Table 1*.

**Table 1:** Demographic characteristics of study participants

Characteristic	Value	Percentage
*Sex, no.*		
Male	78	32%
Female	168	68%
Not specified	1	0.4%
*Age, years*		
< 26	183	74%
26–30	49	20%
> 30	15	6%
Mean (standard deviation)	25 (2.5)	—
Min < median < max	22 < 24 < 36	—
*Cohort*		
A	116	47%
B	131	53%
*Previous experience with VR, no*.		
None	180	73%
0–1 h	44	18%
1–5 h	14	5.7%
5–10 h	4	1.6%
>10 h	5	2.0%
*Modus*		
No guidance	77	31%
Integrated guidance	80	32%
Human guidance	90	36%
*Participation*		
*Active participants*		
No guidance	23	9%
Integrated guidance	26	11%
Human guidance	27	11%
*Observers*		
No guidance	54	22%
Integrated guidance	54	22%
Human guidance	63	26%

### Subjective learning success

**Figure 4:**
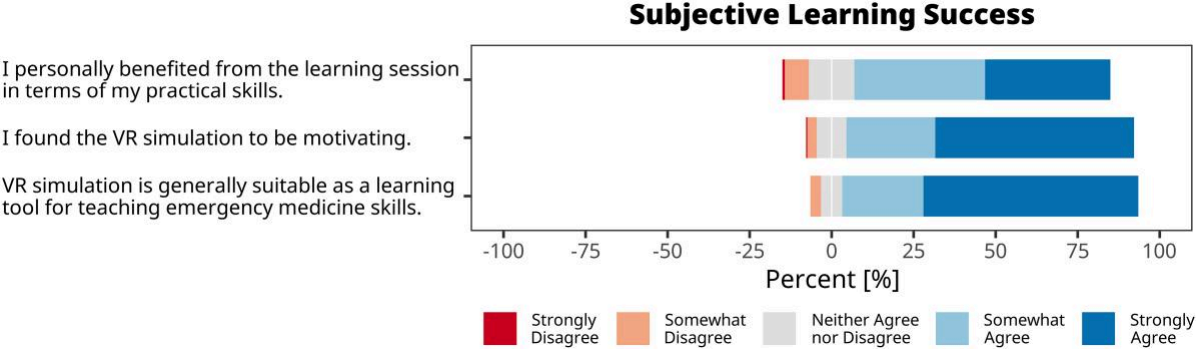
Likert plots (five-point scale) illustrating students’ perceived subjective learning success. Bars represent the percentage of responses for each Likert score.

To evaluate the educational potential of a VR-based training of ACS management, we aimed to assess both subjective and objective learning success. Subjective learning success was measured using a set of three questions in our questionnaire. The results indicated that 90% of students considered the VR simulation a suitable learning tool for teaching emergency medicine skills (*Figure 4*). Additionally, 88% of students found the VR simulation motivating, highlighting its potential to engage learners. Most importantly, 78% of students reported that the simulation session personally benefited them particularly in terms of enhancing their practical skills.

### Objective learning success in virtual reality-based management of acute coronary syndrome/ST-elevation myocardial infarction emergency

**Figure 5:**
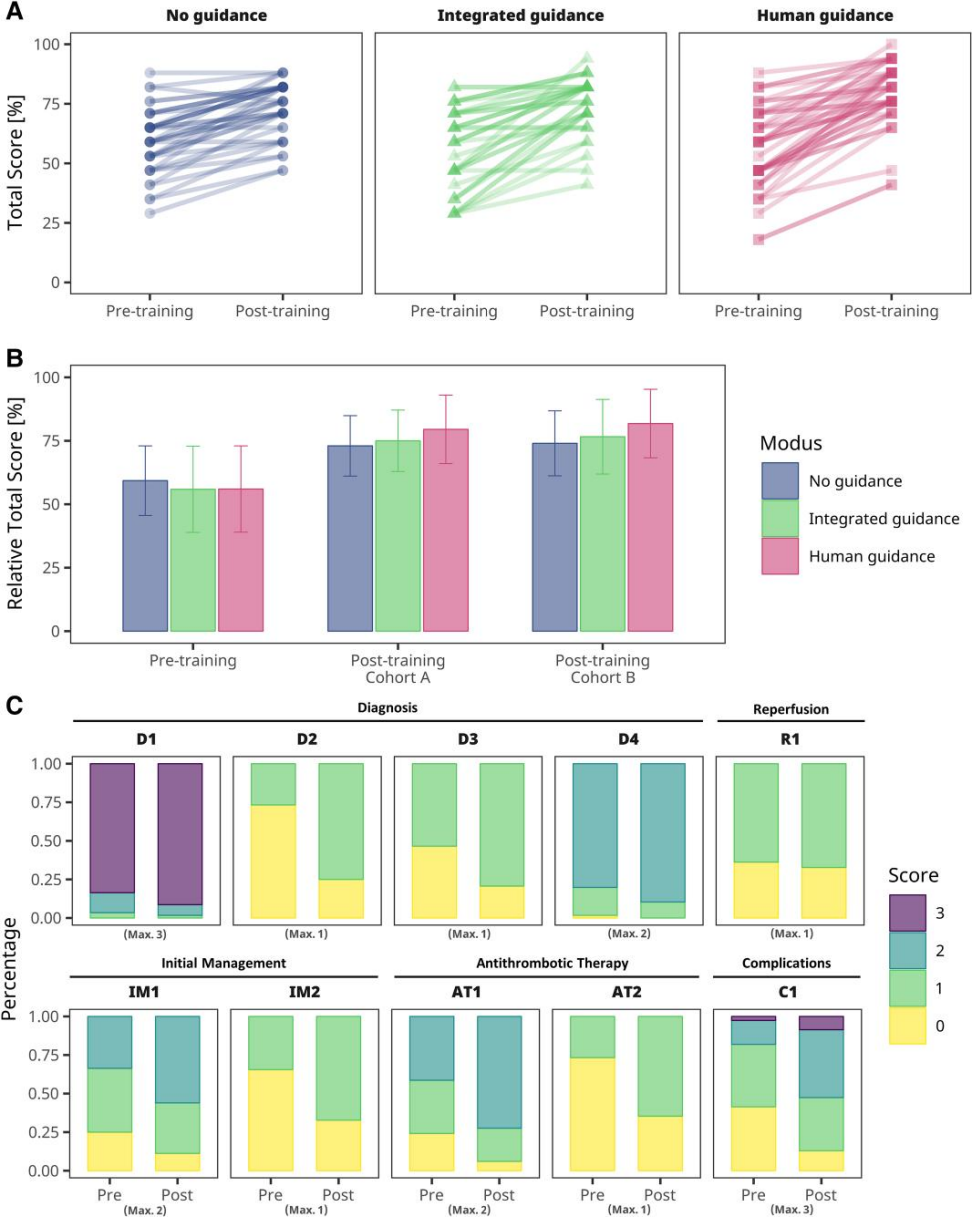
Descriptive results. (*A*) Individual lines representing scores before and after training for Cohort A, with saturation proportional to the number of students with corresponding results. (*B*) Relative total scores pre- and post-training, presented as mean ± SD. (*C*) Relative distribution of scores across all 10 evaluation sheet questions, with maximum score indicated below each plot. For a complete description of the questions, refer to Supplementary material online, *Table S2*.

Across all three seminar modes, nearly all students demonstrated an increase in their total score (*Figure 5A*). However, students in the ‘human guidance’ group showed the greatest improvement, measured as the difference before and after the training.

To account for potential bias from prior exposure to the test questions, the study was conducted with two cohorts. In Cohort A, students completed the evaluation sheets both before and after the seminar, while in Cohort B, students engaged with the sheets only after the training was concluded. Before training, total scores across all groups were similar (*Figure 5B*, Supplementary material online, *Table S5*). After training, total scores increased for both Cohort A and Cohort B. However, the post-training total scores of Cohort A were not higher than those of Cohort B, indicating no significant bias from prior exposure to the test. At the individual question level, the proportion of correct answers increased after training (*Figure 5C*). Notably, the increase in total score was highest in the ‘human guidance’ group, suggesting that active guidance from a tutor enhanced learning outcomes compared to the other modes.

**Figure 6:**
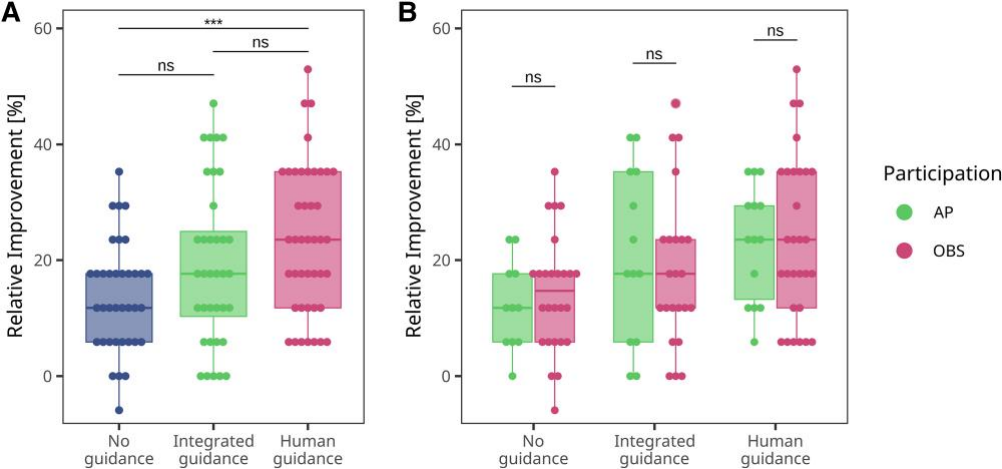
Learning success through VR-based ACS/STEMI training. (*A*) Comparison of relative improvement in total score across the three assisting guidance modes. (*B*) Comparison of total score improvement based on participation type—either actively wearing the VR device and assuming a first-person role (AP, active participant) or passively observing the case development on a screen (OBS, observer) while contributing to discussions and decision-making. Improvement was calculated as the difference between post-training and pre-training total scores (Cohort A). Asterisks indicate significance levels determined using unpaired Mann–Whitney *U* test.

Next, we analysed students’ improvement in more detail to assess learning success in relation to the seminar mode used (*Figure 6A*). Learning success was measured by relative improvement, defined as the difference in total score before and after training, using data from Cohort A.

Across all three groups, students demonstrated a positive learning outcome, improving their scores by at least 14%. However, no significant differences were observed when comparing the ‘integrated guidance’ group (M=19%, SD=14%) with the ‘no guidance’ group (M=14%, SD=9%, W=808.5, P=0.1120) or the ‘human guidance’ group (M=24%, SD=13%) with the ‘integrated guidance’ group (W=629.5, P=0.1520). A statistically significant difference was identified only between the ‘human guidance’ group and the ‘no guidance’ group, W=455.0, P<.001 with students in the ‘human guidance’ group achieving the greatest overall improvement. Additionally, we investigated whether students who were fully immersed in the simulated ACS emergency scenario by wearing the VR headset (active participants, AP) achieved greater learning success than those who attended as passive observers (OBS) (*Figure 6B*). Interestingly, we found no statistically significant difference in learning success between AP and OBS across the three seminar modes (see Supplementary material online, *Table S6*).

**Figure 7:**
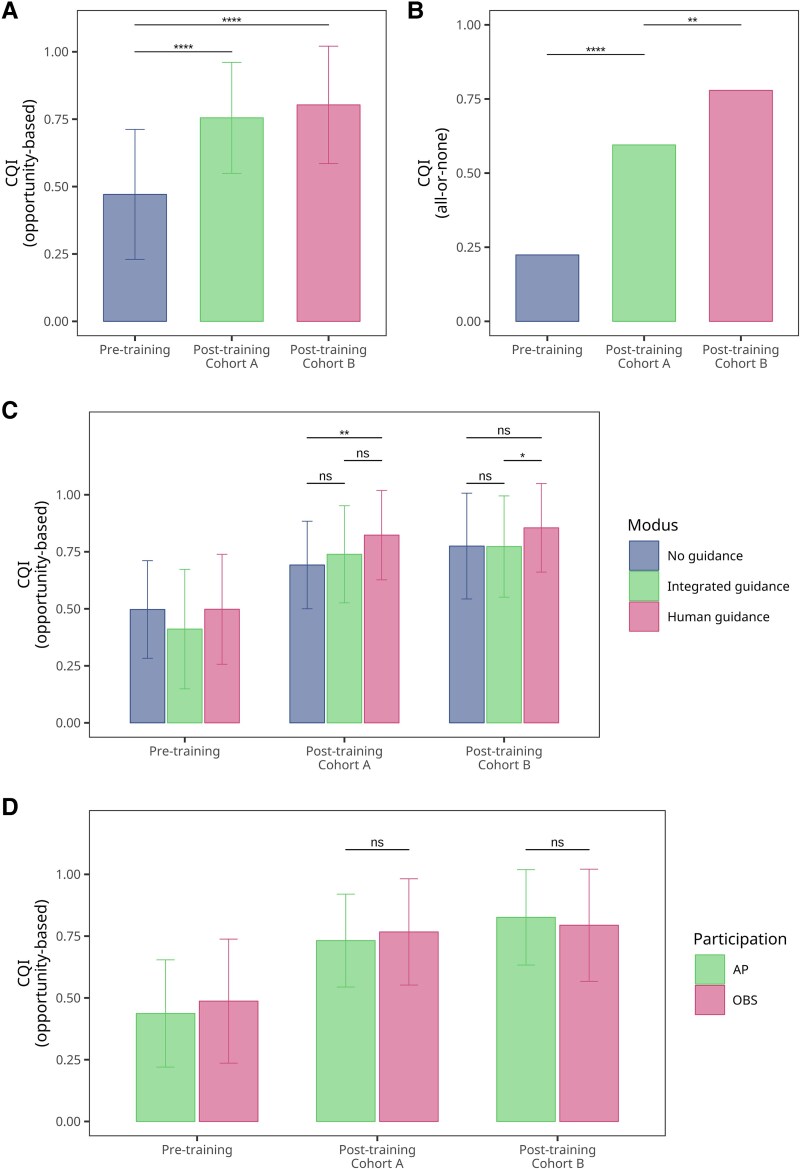
Composite quality indicators (CQI) for evaluation of learning success. (*A–B*) CQIs before and after training for opportunity-based (*A*) and all-or-none CQIs (*B*). (*C–D*) Opportunity-based CQIs compared across guidance modes (*C*) and participation types (*D*). Asterisks indicate significance levels. For details of CQI calculation, refer to Supplementary material online, *Tables S3* and *S4*.

In addition to evaluating the improvement in pure knowledge following the VR-based training, our study aimed to objectively measure enhancements in clinical performance. To assess the quality of care in the management of STEMI patients, we utilized several QIs established by the ESC. These QIs have been extensively tested and shown to correlate with patient outcomes.^4,5,26,27^ Therefore, we adopted the ESC QIs as estimators of students’ potential clinical performance (for a detailed description of QI definitions and calculations, see Supplementary material online, *Tables S3* and *S4*). Among the various QIs and composite QIs (CQIs) developed, the opportunity-based CQI is significantly associated with adjusted mortality and effectively distinguishes different levels of care. Consequently, we focused on CQIs (*Figure 7*). In both Cohorts A (M=0.76, SD=0.21) and B (M=0.80, SD=0.22), the mean opportunity-based CQI increased significantly post-training compared to pre-training (M=0.47, SD=0.24), W=2650.5, P<.001, and W=2411.5, P<.001, respectively (*Figure 7A*). Similarly, the all-or-none CQI also showed a significant increase in Cohorts A (P<.001) and B (P<.001) (*Figure 7B*). At the group level, the opportunity-based CQI demonstrated consistent improvement (*Figure 7C*). While the CQI appeared slightly higher in the ‘human guidance’ group, the results were inconsistent between the two cohorts (see Supplementary material online, *Table S7*). Comparable findings were observed when calculating the all-or-none CQI (see Supplementary material online, *Figure S2A*).

Additionally, we examined whether the mode of participation—AP or OBS, respectively—affected CQI outcomes (*Figure 7D*). Notably, there was no difference in CQI between active participants and observers in Cohorts A and B, P=0.281 and P<0.542, respectively. This finding remained consistent when analysing the all-or-none CQI (see Supplementary material online, *Figure S2B*).

Finally, we assessed individual QIs related to timely reperfusion and adequate P2Y_12_ inhibition. In both groups, these QIs showed a distinct post-training increase (see Supplementary material online, *Figure S2C*–*D*).

## Discussion

In this study involving 247 senior medical students, we investigated whether VR-based emergency training could enhance students’ ability to manage ACS, specifically STEMI, as a critical example.

Students demonstrated a highly positive attitude towards VR for emergency medicine training and reported a subjectively positive learning experience. Similar findings have been reported in previous studies.^23,28–30^ However, while subjective learning success and positive attitudes are important for integrating VR into the medical curriculum, they do not provide a quantitative measure of knowledge improvement—a key limitation of current research on VR-based emergency training.^21,28,31^ We previously demonstrated that integrating VR-based emergency training with STEP-VR is feasible and associated with high perceived learning success.^23^ Building on this, our current study aimed to objectively assess learning outcomes using a pre-post-test design. To control for a potential pre-test sensitization effect, we employed two separate cohorts and found no evidence of a meaningful sensitization effect. Additionally, we examined the impact of different levels of guidance—‘no guidance’, ‘integrated guidance’, and ‘human guidance’—on students’ learning success. Post-training, all three groups showed an improvement in relative scores, with the highest gains observed in the ‘human guidance’ mode. However, the difference between the ‘human guidance’ and ‘integrated guidance’ groups was not statistically significant.

This suggests that for ACS/STEMI training, the integrated learning mode may be as effective as tutor-led seminars, highlighting its potential for self-directed practice. But it has to be acknowledged that our data represent a snapshot after the VR exposure. It can be argued that the repetition of the human tutor might have an effect on the long-term sustainability of the learning success.

To maximize accessibility, it may seem conceivable to implement a system where students can borrow HMDs for individual or small-group training. Increasing user numbers and training frequency could help mitigate the high per-learner cost, which has been a major barrier to widespread VR adoption in medical education.^32^

Notably, students’ mode of participation had no significant effect on their relative improvement. In fact, students who observed the seminar by closely following the simulation on a screen—engaged in the case but not in the primary responsible first-person role—performed no worse than those who actively participated by wearing HMDs, at least in the short term. Therefore, if curricular time is limited, a small-group seminar format—where not every student needs to wear an HMD—may be justified and feasible. This approach would significantly reduce the administrative and financial burden on tutors, the number of required courses per semester cohort, and the demand for HMDs and hosting computers, addressing a major barrier to the widespread implementation of VR into the curriculum.^33,34^ However, this might suggest that the VR immersion might not be crucial for the learning success, and a normal two-dimensional representation would be sufficient. Interestingly, a recent meta-analysis found that less-immersive VR approaches were more effective than fully immersive VR approaches.^35^ Nevertheless, our study design did not allow us to analyse the long-term learning effects after several months. Here, future studies might prove a better outcome for active participation with an immersive VR experience.

Despite the favourable outcomes observed, it is important to acknowledge that knowledge alone, while crucial, may be an insufficient predictor of clinical performance when managing patients.^36,37^ The ultimate goal of medical training is to enhance clinical competence and improve patient outcomes by applying the right knowledge, to the right patient, at the right time, in the right dose. However, the design of our study inherently precluded a direct assessment of clinical performance. Instead, our objective was to evaluate the potential improvement in clinical competence facilitated by VR-based training.

To assess the quality of care in ACS/STEMI management, the ESC has developed and extensively validated QIs for their association with patient outcomes.^2,4,7,8^ Accordingly, we employed ESC QIs as estimators of students’ potential clinical performance. Among these, the opportunity-based composite QI (CQI), which integrates both individual QIs and overall CQIs, has been shown to correlate significantly with adjusted mortality rates and to distinguish different levels of care. Hence, our analysis focused on CQIs. For both cohorts, CQIs were significantly higher post-training, although some variation between cohorts was observed. These differences are likely attributable to natural variation in baseline knowledge and were within an expected range, without affecting the overall trend of improvement. Of note, there was no significant difference between the ‘integrated guidance’ and the ‘human guidance’ groups, reinforcing the notion that the ACS/STEMI emergency scenario within the training application may be suitable for self-directed practice in the absence of a human tutor. Additionally, we found no significant difference between HMD-wearing participants and case-observing participants, suggesting that VR-based emergency training may also benefit students who choose not to fully immerse by actively wearing an HMD, making it feasible for implementation in small-group seminars.

Finally, VR-based ACS/STEMI management training in an ER setting may not be limited to medical students but could also benefit young residents and established physicians, given the high cost and resource demands of conventional simulation-based medical training.^19^ Moreover, this training module might also be valuable for training associated health care professions, such as paramedics and nurses.^38–41^ Looking ahead, well-designed multiplayer versions of immersive simulation training applications may offer additional didactic benefits by facilitating interdisciplinary training that brings together medical students, physicians, nurses, and paramedics. Such an approach could foster collaborative learning and strengthen teamwork and coordination, both essential for the timely management of critically ill patients in emergency medicine, ultimately improving patient outcomes.^4,5^

Although promising, we also need to acknowledge the limitations of this study. Since scoring was conducted by a single, non-blinded rater, we cannot exclude a potential rater bias, although this was partially mitigated by independent verification of random samples. One key limitation is that we could not directly assess students’ performance in a real clinical setting to evaluate the impact on the quality of care and patient outcomes. While QIs may serve as a better predictor than pure knowledge, the adapted QIs used in our study require further validation. Therefore, future research should focus on examining the impact of VR-based emergency training on patient outcomes.^18^ One possible study could involve a supervised phase in the emergency department after the VR-based training to assess the improvement in clinical performance. A more efficient alternative could be using an objective structured clinical examination, which may produce comparable results.

Another limitation is that our study only assessed the short-term effects of VR-based ACS/STEMI management training. Further research is needed to determine if and how VR training influences the development of long-term clinical skills, performance under stress when it really matters, and whether repeated use leads to habituation, potentially altering its effectiveness over time.^21^

Our work supports the findings of a related study by Linder *et al*.^24^ and expands their conclusions with greater generalizability and reduced selection bias, as we included entire student cohorts showing the feasibility of curricular integration and scalability. Additionally, our study examined both a ‘no guidance’ condition and a tutor-moderated ‘human guidance’ condition besides the ‘integrated guidance’ condition, providing deeper insights into the pedagogical value and practical implications of different support formats within VR-based training. Finally, our study adds a novel dimension of clinical performance by incorporating established ESC CQIs, offering an evidence-based proxy for evaluating potential improvements in real-world emergency care delivery.

Taken together, our findings align well with the current literature attesting that VR does seem to be effective in medical education. However, there is still a lack in proving the efficacy of VR to a sufficient level. Additionally, further research is necessary to validate the cost-effectiveness of VR as well as the direct improvement of clinical performance and ultimately patient care.^18,42^

## Conclusions

Medical students and many young physicians are insufficiently prepared to manage critically ill emergency patients presenting with ACS and STEMI, where swift action and precise decision-making are crucial. Based on our findings, VR-based simulation training for managing ACS/STEMI emergencies in a realistic and interactive ER setting appears to be a viable alternative to conventional, resource-intensive simulation-based education. We believe our findings have broader implications, supporting the idea that VR-based ACS/STEMI training could be valuable not only for medical students but also as an effective tool for residents and other healthcare professionals. Future research should explore the long-term improvements of VR as well as the impact on clinical performance.

By lowering the cost and labour demands of traditional simulations, this approach could enable more scalable and frequent refresher training, ultimately contributing to better patient care and outcomes.

## Supplementary Material

ztaf094_Supplementary_Data

## Data Availability

All relevant data are reported in the article. Additional data can be provided by the corresponding author on reasonable request.
