# Supplementary material for: Effectiveness of fully immersive virtual reality-based simulation training on objective knowledge acquisition in acute coronary syndrome/ST-elevation myocardial infarction emergency management: a pre-post-intervention study
Source: Eur Heart J Digit Health. 2025 Sep 4;7(1):ztaf094. doi: 10.1093/ehjdh/ztaf094 (PMC12821071; doi:10.1093/ehjdh/ztaf094)
Supplement: ztaf094_Supplementary_Data [file ztaf094_supplementary_data.docx]

Supplementary Material

**Effectiveness of Fully Immersive VR-Based Simulation Training on Objective Knowledge Acquisition in ACS/STEMI Emergency Management: A Pre-Post Intervention Study**


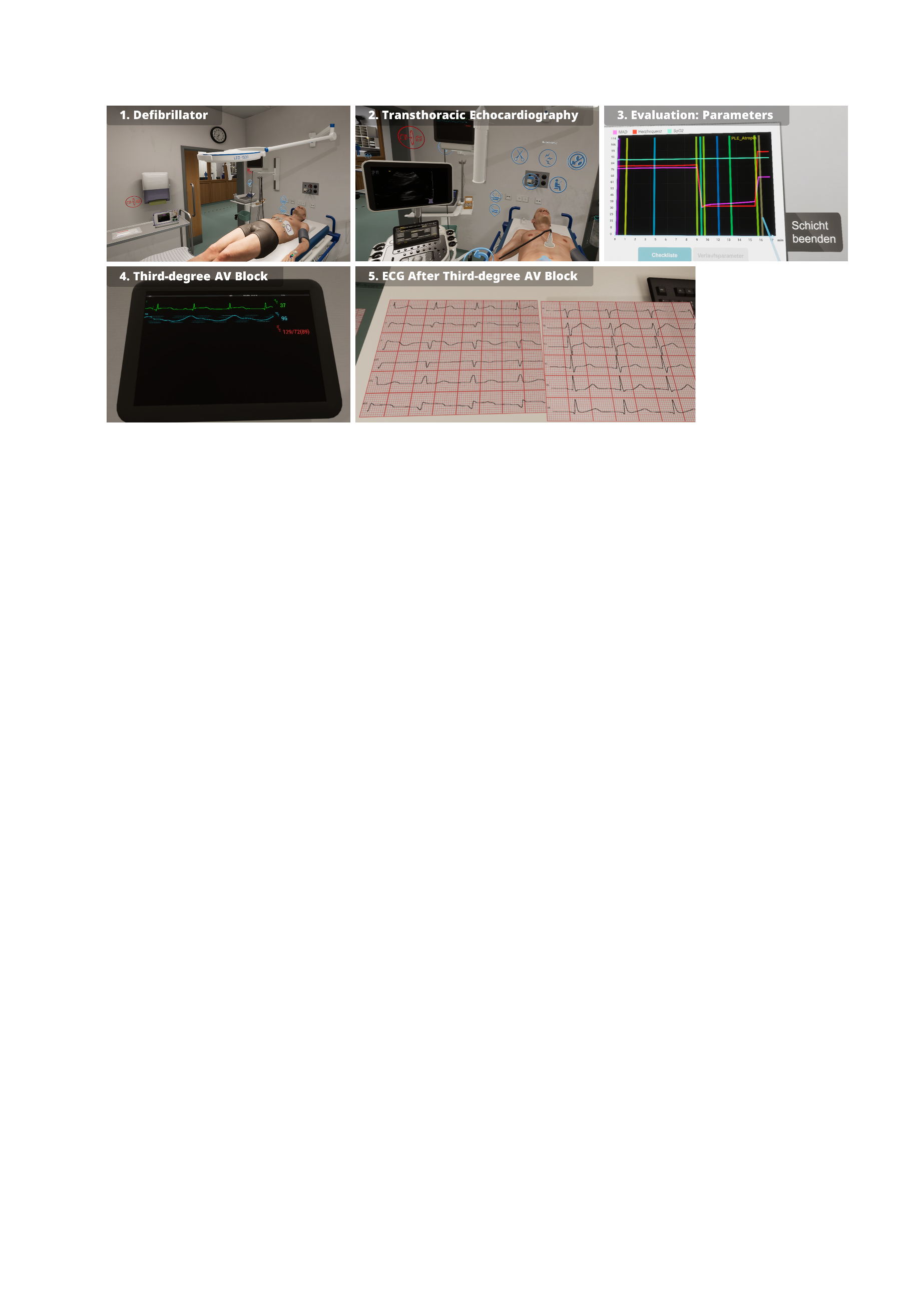


Supplement Figure 1 | Additional screenshots. Application of external defibrillator (1), optional transthoracic echocardiography (2), progressive parameters as part of the performance evaluation (3), monitoring (4) and ECG after third-degree AV block are depicted.

Supplementary Table 1 | Design of the questionnaire.

| **Category** | **Question type** | **Question text** |
| --- | --- | --- |
| Demographics | Open-ended  Ordinal (5 levels)  Nominal | Age:  Gender:  How often do you play first-person computer games?  How much experience do you have with virtual reality applications?  Did you work on the scenario yourself in virtual reality (Active Participant) or were you involved as an observer? |
| Subjective learning success | Likert (5 point) | VR simulation is generally suitable as a learning tool for teaching emergency medicine skills.  I personally benefited from the learning session in terms of my practice skills.  I found the VR simulation to be motivating. |

Supplementary Table 2 | Design of the evaluation sheets.

| **Questions text** | **ESC reference** | | | **Max. Score** | **Scoring** |
| --- | --- | --- | --- | --- | --- |
|  | **Class** | **Level** | |  |  |
| ***Diagnosis*** | | | | | |
| Name three symptoms ("red flags") that may indicate myocardial ischemia. | -* | | -* | 3 | 1 point for each correct symptom group (max. 3 points):   - acute chest pain (retrosternal, radiating) - vegetative symptoms (sweating, nausea, vomiting, anxiety, agitation) - symptoms of acute cardiac insufficiency (dyspnea, vertigo, disturbance of consciousness, hypotension, cardiac asthma…) - arrythmias (tachycardia, bradycardia, palpitations) |
| In the case of a possible acute coronary syndrome, after what time should a 12-lead ECG have been written? | I | | B | 1 | - max. target delay of 10 min from first medical contact |
| What limb and/or chest wall leads are used to recognize inferior ST elevation infarction? | -* | | -* | 1 | - II, III and aVF leads |
| You have a suspected acute coronary syndrome. Which serum markers give you information after blood sampling? Name the two most clinically relevant. | I | | C | 2 | 1 point for each correct answer:   - (cardiac) troponin (T/I) - creatine kinase (CK/CK-MB) |
| ***Initial Management*** | | | | | |
| Which concomitant medications should be administered on a symptom-oriented basis? Name two drugs or agents. | IIa** | | C** | 2 | 1 point for each correct answer out of (max. 2 points):   - analgesic: opioid analgesics (e.g., morphine) - antiemetic: dimenhydrinate - anxiolytic: mild tranquillizer (usually a benzodiazepine) |
| When is the administration of oxygen indicated in acute ST elevation myocardial infarction? | I | | C | 1 | - SaO_2_ <90% (PaO_2_ <60 mmHg) |

* Not part of the evidence-based recommendation of the ESC guideline, but important to know for diagnosis of STEMI. ** ESC reference refers to analgesic and anxiolytic medication as antiemetic therapy is not discussed in the guidelines.

Supplementary Table 2 | *(continued).*

| **Questions text** | **ESC reference** | | **Max. Score** | **Scoring** |
| --- | --- | --- | --- | --- |
|  | **Class** | **Level** |  |  |
| ***Reperfusion Therapy*** | | | | |
| What is the most urgent therapeutic measure in ST-segment elevation myocardial infarction? | I | A | 1 | - primary percutaneous coronary intervention |
| ***Antithrombotic Therapy*** | | | | |
| Which two therapeutically effective drugs can already be administered in cases of high-grade suspected acute coronary syndrome? | I | B/C | 2 | 1 point for each correct medication:   - aspirin (ASA) - unfractionated heparin |
| In addition to the two standards, which antithrombotic drug or class of drugs is indicated when a diagnosis of myocardial infarction is confirmed? | I | A | 1 | - second antiplatelet drug: potent P2Y12 inhibitor (prasugrel or ticagrelor) or clopidogrel if not available |
| ***Management of Complications*** | | | | |
| Which therapeutic measures are acutely useful for bradyarrhythmia affecting the circulation (e.g., higher-grade AV block)? | I | C | 3 | 1 point for each correct medication:  positive chronotropic medication with:   - i.v. atropine and/or - i.v. adrenaline   1 point for:   - temporary pacing is indicated in cases of failure to respond to positive chronotropic medication |

Supplementary Table 3 | Calculation of the Quality Indicators (QI).

| **Domain of Care** | **Definition of QI** | **Corresponding QI calculation** |
| --- | --- | --- |
| Reperfusion-Invasive Strategy | **Main QI2 (STEMI) (timely reperfusion):**  Proportion of patients with timely reperfusion. Timely is defined as:  For patients treated with primary PCI and admitted to centers with catheterization laboratory facilities: <60 min from door-to-arterial access for reperfusion with PCI  **Numerator**: number of STEMI patients treated with primary PCI within the above delays.  **Denominator**: all STEMI patients eligible for reperfusion by primary PCI (onset of symptoms to diagnosis  **Method of reporting**: proportion (standard error) | **Main QI2 (STEMI) (timely reperfusion):**  Proportions of students with completely correct answers of questions:   - D2 (time frame for ECG monitoring) - D3 (leads for STEMI diagnosis) - R1 (reperfusion strategy   **Numerator**: number of students with completely correct answers.  **Denominator**: number of all students participating  **Method of reporting**: proportion |
| Anti-thrombotics during Hospitalization | **Main QI4 (adequate P2Y_12_ inhibition):**  Proportion of patients with ‘adequate P2Y_12_ inhibition’  **Numerator**: number of STEMI patients with ‘adequate P2Y_12_ inhibitor’ at discharge.  **Denominator**: STEMI patients alive at discharge and without contraindications to P2Y_12_ inhibitors  **Method of reporting**: proportion (standard error) | **Main QI4 (adequate P2Y_12_ inhibition):**  Proportion of students with completely correct answers of question:   - AT2 (P2Y_12_ inhibitor)   **Numerator**: number of students with completely correct answers.  **Denominator**: number of all students participating  **Method of reporting**: proportion |

Supplementary Table 4 | Calculation of the composite Quality Indicators (CQI).

| **Domain of Care** | **Definition of QI** | **Corresponding QI calculation** |
| --- | --- | --- |
| Composite QI | **Main composite QI (opportunity based)**  Proportion of care processes received for each patient; care processes are [only relevant mentioned]:   - Proportion of patients reperfused among eligible (STEMI with FMC<12 h after onset of pain) - Low dose aspirin (unless high bleeding risk or oral anticoagulation) - Adequate P2Y_12_   **Numerator**: number of times particular care processes were performed  **Denominator**: number of opportunities the patient had to receive that process or the number of opportunities the hospital had to provide the process (i.e., number of applicable care processes)  **Method of reporting**: mean (standard deviation) | **Main composite QI (opportunity based)**  Proportion of complete correct answers for each student:   - D2 (time frame for ECG monitoring) - D3 (leads for STEMI diagnosis) - R1 (reperfusion strategy) - AT1 (mentioning of ASS) - AT2 (P2Y_12_ inhibitor)   **Numerator**: number of complete correct answers  **Denominator**: number of applicable answers (i.e., total number of questions mentioned above)  **Method of reporting**: mean (standard deviation) |
|  | **Secondary composite QI (all-or-none)**  Proportion of patients receiving all of the following treatments:   - Low-dose aspirin - P2Y12 inhibitor (unless documented contraindication)   **Numerator**: number of patients receiving all treatments  **Denominator**: number of all patients  **Method of reporting**: proportion | **Secondary composite QI (all-or-none)**  Proportion of students answering all of the following correct:   - AT1 (mentioning of ASS) - AT2 (P2Y_12_ inhibitor)   **Numerator**: number of students answering all questions correctly  **Denominator**: number of all students participating  **Method of reporting**: proportion |

**Supplementary Table 5 | Improvement of relative total score based on guidance mode.**

| **Round** | **Group** | $\boldsymbol{M}$ **[%]** | $\boldsymbol{SD}$ **[%]** | $\boldsymbol{n}$ |
| --- | --- | --- | --- | --- |
| Pre-training | No guidance | 59 | 13.7 | 37 |
| Pre-training | Integrated guidance | 56 | 17.0 | 36 |
| Pre-training | Human guidance | 56 | 17.0 | 43 |
| Post-training Cohort A | No guidance | 73 | 11.9 | 37 |
| Post-training Cohort A | Integrated guidance | 75 | 12.1 | 36 |
| Post-training Cohort A | Human guidance | 80 | 13.5 | 43 |
| Post-training Cohort B | No guidance | 74 | 12.8 | 40 |
| Post-training Cohort B | Integrated guidance | 77 | 14.7 | 44 |
| Post-training Cohort B | Human guidance | 82 | 13.5 | 46 |

**Supplementary Table 6 | Statistical results for the comparison of total score improvement based on participation type.**

| ***Descriptive Statistics*** | | | | | | | |
| --- | --- | --- | --- | --- | --- | --- | --- |
| **Group** | **Participation** | | $\boldsymbol{M}$ **[%]** | $\boldsymbol{SD}$ **[%]** | | $\boldsymbol{n}$ | |
| No guidance | AP | | 12 | 1.30 | | 11 | |
| No guidance | OBS | | 14 | 1.70 | | 26 | |
| Integrated guidance | AP | | 21 | 2.54 | | 13 | |
| Integrated guidance | OBS | | 18 | 2.25 | | 23 | |
| Human guidance | AP | | 23 | 1.69 | | 14 | |
| Human guidance | OBS | | 24 | 2.38 | | 29 | |
| ***Statistical Results*** | | | | | | | |
| **Group 1** | | **Group 2** | | | $\boldsymbol{W}$ | | $\boldsymbol{p}$ |
| ***No guidance*** | | | | | | | |
| Active participant | | Observer | | | 127.0 | | 0.599 |
| ***Integrated guidance*** | | | | | | | |
| Active participant | | Observer | | | 165.0 | | 0.618 |
| ***Human guidance*** | | | | | | | |
| Active participant | | Observer | | | 204.5 | | 0.979 |

AP, active participant; OBS, observer.

**Supplementary Table 6 | Statistical results for the comparison of opportunity-based CQIs by modus.**

| ***Descriptive Statistics*** | | | | | | | |
| --- | --- | --- | --- | --- | --- | --- | --- |
| **Timepoint** | **Group** | | $\boldsymbol{M}$ | | $\boldsymbol{SD}$ | | $\boldsymbol{n}$ |
| Pre-training | No guidance | | 0.50 | | 0.21 | | 37 |
|  | Integrated guidance | | 0.41 | | 0.26 | | 36 |
|  | Human guidance | | 0.50 | | 0.24 | | 43 |
| Post-training  Cohort A | No guidance | | 0.69 | | 0.19 | | 37 |
|  | Integrated guidance | | 0.74 | | 0.21 | | 36 |
|  | Human guidance | | 0.82 | | 0.20 | | 43 |
| Post-training  Cohort B | No guidance | | 0.78 | | 0.23 | | 40 |
|  | Integrated guidance | | 0.77 | | 0.22 | | 44 |
|  | Human guidance | | 0.86 | | 0.19 | | 47 |
| ***Statistical Results*** | | | | | | | |
| **Group 1** | | **Group 2** | | $\boldsymbol{W}$ | | $\boldsymbol{p}$ | |
| ***Post-training Cohort A*** | | | | | | | |
| No guidance | | Integrated guidance | | 574 | | 0.288 | |
| No guidance | | Human guidance | | 495 | | 0.002 | |
| Integrated guidance | | Human guidance | | 595.5 | | 0.066 | |
| ***Post-training Cohort B*** | | | | | | | |
| No guidance | | Integrated guidance | | 893 | | 0.906 | |
| No guidance | | Human guidance | | 731.5 | | 0.058 | |
| Integrated guidance | | Human guidance | | 799 | | 0.047 | |

**
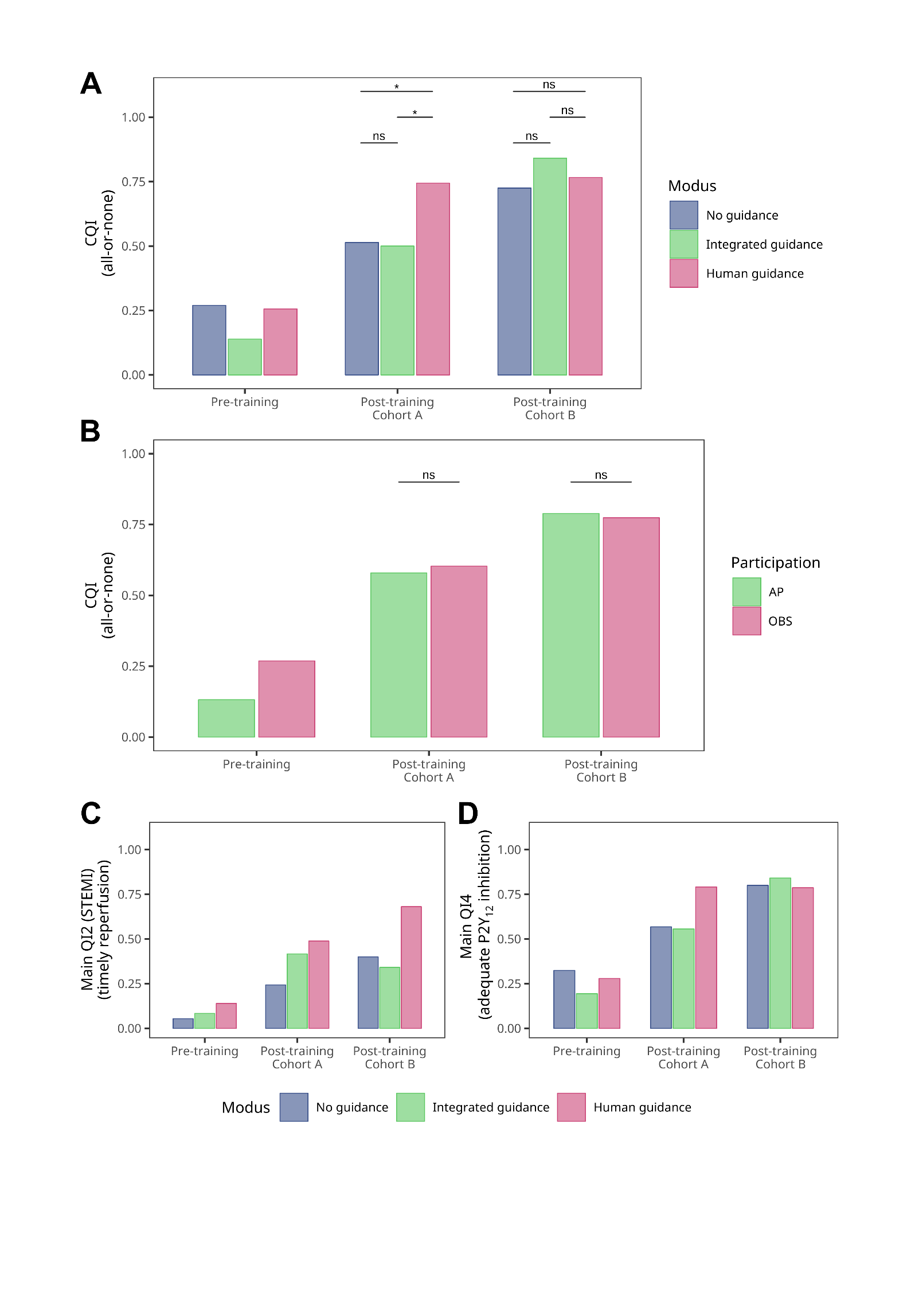
**

Supplemental Figure 2 | Additional quality indicators for evaluation of learning success. A-B, all-or-none CQIs tested between modus (A) and participation (B). C-D, single QI for timely reperfusion (C) and adequate P2Y12 inhibition (D). Stars indicate significance.
